# New isolations of the rabies-related Mokola virus from South Africa

**DOI:** 10.1186/s12917-017-0948-0

**Published:** 2017-01-31

**Authors:** Jessica Coertse, Wanda Markotter, Kevin le Roux, Daniel Stewart, Claude T. Sabeta, Louis H. Nel

**Affiliations:** 1 Center for Viral Zoonoses, Department of Medical Virology, School of Medicine, Faculty of Health Sciences, University of Pretoria, Pretoria, 0001 South Africa; 2 Allerton Provincial Veterinary Laboratory, Pietermaritzburg, KwaZulu-Natal 3200 South Africa; 3 Department of Agriculture and Environmental Affairs, KwaZulu-Natal Rabies Project, Pietermaritzburg, KwaZulu-Natal South Africa; 4 Agricultural Research Council-Onderstepoort Veterinary Institute (ARC-OVI), Pretoria, 0110 South Africa; 5 Department of Microbiology and Plant Pathology, Faculty of Natural and Agricultural Sciences, University of Pretoria, Pretoria, 0001 South Africa

**Keywords:** Mokola virus, Lyssavirus, Rabies-related, South Africa

## Abstract

**Background:**

Mokola virus (MOKV) is a rabies-related lyssavirus and appears to be exclusive to the African continent. Only 24 cases of MOKV, which includes two human cases, have been reported since its identification in 1968. MOKV has an unknown reservoir host and current commercial vaccines do not confer protection against MOKV.

**Results:**

We describe three new isolations of MOKV from domestic cats in South Africa. Two cases were retrospectively identified from 2012 and an additional one in 2014.

**Conclusions:**

These cases emphasize the generally poor surveillance for rabies-related lyssaviruses and our inadequate comprehension of the epidemiology and ecology of *Mokola lyssavirus* per se.

**Electronic supplementary material:**

The online version of this article (doi:10.1186/s12917-017-0948-0) contains supplementary material, which is available to authorized users.

## Background

The *Lyssavirus* genus currently consists of 14 recognized species all capable of causing rabies, a fatal encephalitic disease. The prototype species of this genus is *Rabies lyssavirus* and the rest are known as the rabies-related lyssaviruses [1]. Mokola virus (MOKV), a rabies-related lyssavirus, was first isolated from shrews in the Mokola forest in Nigeria in the late 1960s [2, 3]. Shortly thereafter, MOKV was reported as the causative agent of a neurological disease in two children from Nigeria [4, 5]. However, these reports remain controversial for a variety of reasons. The first isolation was made from the cerebrospinal fluid of a 3.5-year-old girl in 1968. The girl recovered without any neurological sequelae while no virus neutralizing antibodies (VNAs) were detected [4]. This is unexpected as reports of patients surviving rabies are exceptionally rare. In the majority of survivor cases the patient is burdened with moderate to severe neurological sequelae [6, 7] with VNAs regarded as a primary mechanism of viral clearance [6, 8]. The second human isolate was obtained from the brain tissue of a 6-year-old girl in 1971 that died from suspected meningitis or encephalitis. In both these human cases clinical symptoms were atypical for classical rabies virus (RABV) infection [5]. It is doubtful that these isolates are still in existence and no genetic information is available [9].

Up until 2013, only 18 confirmed MOKV isolations, from a variety of mammalian host species including shrews, cats, dogs and a rodent (*Lophuromys sikapusi*), were known to exist [9]. MOKV was first encountered in South Africa in 1970, when the virus was isolated from a domestic cat in the KwaZulu-Natal (KZN) province [10]. In 1981, it became evident that the available rabies vaccines did not confer protection against MOKV when this virus was isolated from vaccinated cats and a dog in Zimbabwe [11]. It appears that MOKV is exclusive to the African continent and all isolations for the last 20 years have been made from southern Africa. Little is known about the epidemiology of this lyssavirus and the problem is compounded by an unknown reservoir and limited surveillance throughout the continent. In the few instances where rabies diagnostic facilities are available and operational in Africa, only the fluorescent antibody test (FAT) is used. The FAT relies on the use of a polyclonal fluorescein isothiocyanate conjugated anti-lyssavirus globulin that is capable of detecting all known lyssavirus species but cannot distinguish between them. As a result, positive cases are reported as rabies but the actual causative lyssavirus species is rarely identified. In South Africa, where molecular characterization of FAT-positive samples is frequently done, the majority of MOKV isolations have been from the KZN (*n*=4) and Eastern Cape (EC) provinces (*n*=5) with a single isolation reported from the Mpumalanga province. These isolates differ by between 0–5.7% and 0–2% at the nucleotide and amino acid levels of the nucleoprotein (N) gene. However, comparison of the N-gene of all known MOKV isolates demonstrated variations of up to 15% and 6.4% on the nucleotide and amino acids levels respectively [9].

Rabies virus (RABV) vaccines do not protect against MOKV [9, 11, 12] and it should be appreciated that the domestic cat is the most commonly MOKV-infected host species. The frequent contact between cats and their owners suggests a potential risk of spill-over infection to humans. Considering this situation, it is clear that a better understanding of the incidence and ecology of MOKV ecology is of utmost importance. Here we report the isolation and characterization of three MOKV isolations from cats from KZN, South Africa, 4 years after the last isolation of this virus in 2008 from South Africa. The first case was identified in January 2014 and subsequently a small retrospective study on selected samples was undertaken, identifying an additional two cases from 2012.

## Methods

### The study

In January 2014, a private veterinarian submitted brain material from a domestic cat that had died of suspected rabies in Pietermaritzburg, KZN, South Africa (laboratory reference number: 14/024) to the Allerton Provincial Veterinary Laboratory. The FAT was performed by staining acetone-fixed impression smears of the brain material with a polyclonal fluorescein isothiocyanate conjugated anti-lyssavirus globulin (OIE Rabies Reference Laboratory of the Agricultural Research Council-Onderstepoort Veterinary Institute (ARC-OVI), South Africa) [13]. Lyssavirus antigen was observed, but in this specific case the staining was of dull fluorescence clearly atypical of rabies virus positive samples. Such atypical staining which has previously been noted in MOKV infections prompted further investigation of this case. The positive FAT result was confirmed at the OIE Rabies Reference Laboratory at Onderstepoort (ARC-OVI, South Africa) and the sample genetically characterized at the University of Pretoria. Following the subsequent identification of sample 14/024 as MOKV, it was decided to investigate and molecularly characterize other FAT positive rabies samples from domestic animals from throughout KZN. Selected archived samples (*n*=36), were characterized by nucleotide sequencing resulting in the identification of an additional two MOKV cases.

### RNA extraction, RT-PCR and phylogenetic analysis

Total RNA was isolated from brain material using Trizol reagent (Invitrogen) according to the manufacturer’s instructions. The complete N-, phosphoprotein- (P), matrix protein- (M) and glycoprotein (G) genes were sequenced using different primer combinations and cycling conditions (Additional file 1: Table S1) for all MOKV samples. Briefly, reverse transcription was performed for all samples using the following protocol: 10 pmol of forward primer was added to 5 μl total RNA and incubated at 94 °C for 1 min. These reactions were cooled on ice for 5 min followed by reverse transcription for 90 min at 42 °C in a final volume of 20 μl containing 1 x reverse transcriptase buffer (containing 250 mM Tris-HCl, 40 mM MgCl_2_, 150 mM KCl, 5 mM dithiothreithol, Roche), 2.2 μl dNTP mix (10 mM, Promega), 8 U Avian myeloblastosis virus reverse transcriptase (Roche) and 16 U Recombinant RNasin Ribonuclease inhibitor (Promega). The genes were subsequently amplified using 20 μl cDNA in a final volume of 100 μl containing 1 x DreamTaq Buffer (containing KCl, (NH4)_2_SO_4_ and 20 mM MgCl_2_, Thermo Scientific), 10 pmol of forward primer (Additional file 1: Table S1), 12.5 pmol reverse primer (Additional file 1: Table S1) and 1.25 U DreamTaq DNA polymerase (Thermo Scientific). Analysis and sequencing of PCR products were performed as described previously [14]. Nucleotide sequences were edited and assembled using BioEdit Sequence Alignment Editor Version 7 [15]. The partial N-gene for RABV sequences (Additional file 2: Table S2) or concatenated and individual genes for MOKV sequences (Additional file 3: Table S3) was analyzed with jModeltest 2 [16] for each dataset to determine the most appropriate substitution model. Data was analyzed using BEAST v.1.8 [17] using a random starting tree with a strict clock for each dataset and assuming an exponential population growth with Markov Chain Monte Carlo (MCMC) chains of 50 million generations.

### Virus isolation

Virus was isolated from MOKV samples on murine neuroblastoma cells as described previously [18].

## Results

**Fig. 1:**
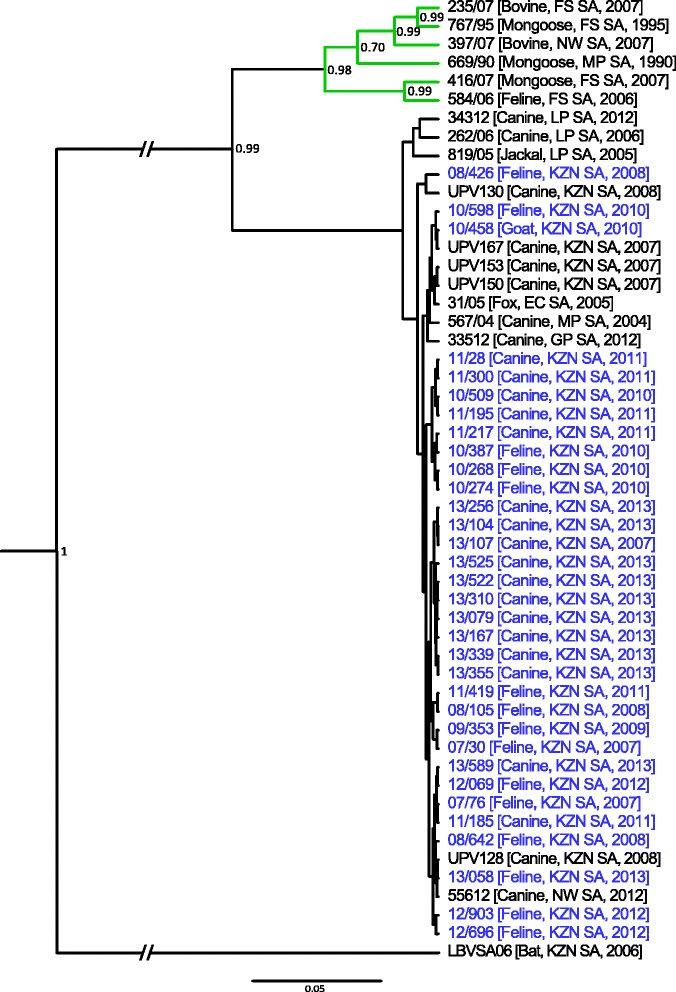
Bayesian analysis of the partial N-gene sequences (540 bp) of the 33 archival samples and other rabies virus sequences from South Africa (Additional file 2: Table S2) applying the general time reversible substitution model with gamma distribution. Laboratory reference numbers are shown for all sequences, followed by the host species, country of origin (EC SA: Eastern Cape province, South Africa; FS SA: Free State province, South Africa; GP SA: Gauteng province, South Africa; KZN SA: Kwa-Zulu Natal province, South Africa; LP SA: Limpopo province, South Africa; MP SA: Mpumalanga province, South Africa; NW SA: North West province, South Africa) and year. All rabies virus sequences determined in this study are indicated in blue

The partial N-gene sequences (using primers 001lys/550B, Additional file 1: Table S1) [19] of the 36 samples were determined and subsequent to the analyses two more MOKV cases were identified i.e. 12/458 and 12/604 (Table 1). Based on partial N-gene sequences, the remaining 33 archival samples were determined to be the canid variant of RABV. Bayesian analysis indicated that all new RABV sequences group with other RABV sequences from the same time period (Fig. 1).

**Table 1:** Information of domestic animals from the KwaZulu-Natal province submitted for molecular characterization

Laboratory reference number	Host	Collection area	Date	Genbank accession number (gene)
07/30	*Felis catus* (feline)	Empangeni	11/01/2007	KP994621
07/76	*Felis catus* (feline)	Melmoth	29/01/2007	KP994616
08/105	*Felis catus* (feline)	Kwadukuza	18/02/2008	KP994617
08/426	*Felis catus* (feline)	Jozini	03/07/2008	KP994618
08/642	*Felis catus* (feline)	Exodondakukuska	15/10/2008	KP994619
09/353	*Felis catus* (feline)	Nkambanana	07/08/2009	KP994620
10/268	*Canis familiaris* (canine)	Umdoni	20/05/2010	KJ744302
10/274	*Canis familiaris* (canine)	Hibiscus coast	24/05/2010	KJ744308
10/387	*Canis familiaris* (canine)	Umzumbe	23/08/2010	KJ744303
10/458	*Capra aegagrus hircus* (goat)	Umzimkulu	29/09/2010	KJ744309
10/509	*Canis familiaris* (canine)	Mkhambathini	22/10/2010	KJ744304
10/598	*Felis catus* (feline)	Dundee	16/11/2010	KP994606
11/28	*Canis familiaris* (canine)	Richmond	14/01/2011	KP994597
11/185	*Canis familiaris* (canine)	Mkahambathini	24/03/2011	KJ744305
11/195	*Canis familiaris* (canine)	Mkhambathini	28/03/2011	KP944598
11/217	*Canis familiaris* (canine)	Umdoni	12/04/2011	KJ744310
11/300	*Canis familiaris* (canine)	Richmond	30/05/2011	KJ744307
11/419	*Felis catus* (feline)	Ethekweni	16/08/2011	KP994607
12/069	*Felis catus* (feline)	Okhalamba	31/01/2012	KP994610
12/458	*Felis catus* (feline)	Durban	13/06/2012	KP899610(N), KP899619(P), KP899613(M), KP899616(G)
12/604	*Felis catus* (feline)	Durban	08/07/2012	KP899611(N), KP899620(P), KP899614(M), KP899617(G)
12/696	*Felis catus* (feline)	Ethekweni	27/07/2012	KP994608
12/903	*Felis catus* (feline)	Okhahlamba	02/10/2013	KP994609
13/058	*Felis catus* (feline)	Ethekweni	25/01/2013	KP994611
13/079	*Canis familiaris* (canine)	Umlazi	01/02/2013	KP994601
13/104	*Canis familiaris* (canine)	Amanzimtoti	14/02/2013	KP994599
13/107	*Canis familiaris* (canine)	Westville	18/02/2013	KP994602
13/167	*Canis familiaris* (canine)	Adams mission	18/03/2013	KP994612
13/256	*Canis familiaris* (canine)	Amanzimtoti	09/05/2013	KP994613
13/310	*Canis familiaris* (canine)	Umkomaas	10/06/2013	KP994603
13/339	*Canis familiaris* (canine)	Umlazi	21/06/2013	KP994614
13/355	*Canis familiaris* (canine)	Amanzimtoti	28/06/2013	KP994615
13/522	*Canis familiaris* (canine)	Athlone Park	27/09/2013	KP994604
13/525	*Canis familiaris* (canine)	Lewis Drive	30/09/2013	KP994605
13/589	*Canis familiaris* (canine)	Uthukela	29/10/2013	KP994600
14/024	*Felis catus* (feline)	Pietermaritzburg	09/01/2014	KP899612(N), KP899621(P), KP899615(M), KP899618(G)

Details and clinical history of the cats that tested positive for MOKV are summarized in Table 2. For all the MOKV cases, the complete sequences of four structural genes (i.e. N-, P-, M- and G genes) were determined.

**Table 2:** Details and clinical history of cats from the KwaZulu-Natal province, South Africa that tested positive for Mokola virus

	Laboratory reference number		
	14/024	12/458	12/604
Location	Pietermaritzburg	Durban	Durban
Host details	Male, 4-year-old	Female, 1-year-old	Female, 1-year-old
Clinical signs	Fever (>40 °C), ataxia convulsions	Fever (39.6 °C), ataxia, decreased appetite	Growling, aggression, ataxia
Clinical duration	8-9 January 2014	10-13 June 2012	6-8 July 2012
Clinical outcome	Died of the disease	Euthanized after collapsing and being non-responsive	Euthanized after being non-responsive

**Fig. 2:**
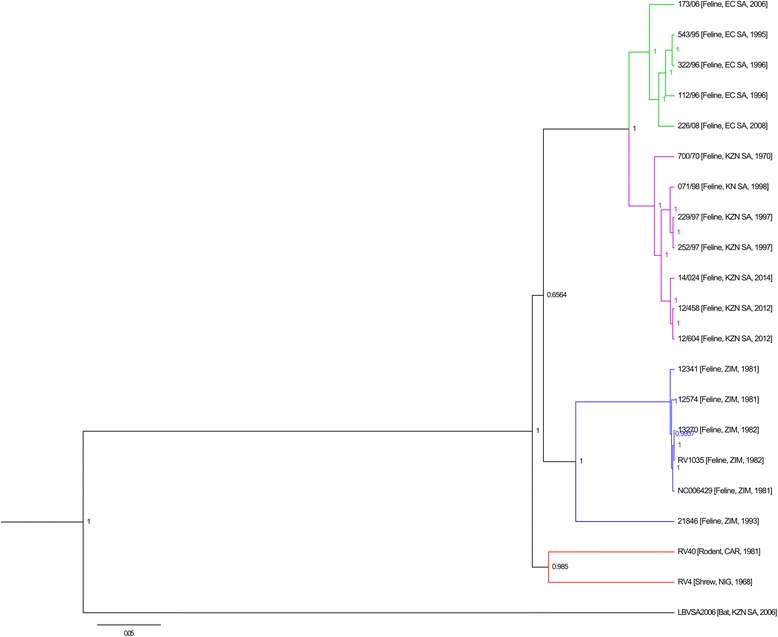
Bayesian analysis of the concatenated coding region of the N-, P-, M- and G-genes of all Mokola virus isolates (Additional file 3: Table S3) applying the general time reversible substitution model with gamma distribution and invariable sites. Laboratory reference numbers are shown for all sequences, followed by the host species, country of origin (KZN SA: KwaZulu-Natal province, South Africa; EC SA: Eastern Cape province South Africa; ZIM: Zimbabwe; CAR: Central African Republic; NIG: Nigeria) and year of isolation

For all the MOKV cases, the complete coding region sequence of four structural genes (i.e. N-, P-, M-, and G-genes) were determined. Each dataset (concatenated or individual genes) were analysed using a Bayesian approach. The tree topology (Fig. 2) observed was similar irrespective of the gene used for analysis (Additional file 4: Figure S1, Additional file 5: Figure S2, Additional file 6: Figure S3, Additional file 7: Figure S4) with the exception of the M-gene. For the M-gene dataset (Additional file 6: Figure S3), isolate RV4 (from Nigeria) grouped with an isolate from Zimbabwe (posterior probability = 0.8144) and not with the isolate from the Central African Republic as observed for the other datasets. This support findings from previous studies that MOKV phylogeny is strongly influenced by geographical derivation [9]. For the isolates from South Africa, two separate clusters were found to represent isolates from the two provinces viz. KZN and EC. Within the KZN cluster, the new MOKV isolates form a well-supported new clade. Nucleotide divergence of all known MOKV isolates (Additional file 8: Table S4, Additional file 9: Table S5, Additional file 10: Table S6 and Additional file 11: Table S7) ranged from 0.1-13.9%, 0-25.2%, 0-17.8% and 0.2-19.6% for the N-, P-, M- and G-genes respectively.

## Discussion

There is no active surveillance for rabies-related lyssaviruses in Africa and subsequently the epidemiology of these viruses remains obscure. Rabies, caused by RABV, is endemic in KZN, South Africa and has been tackled through mass vaccination campaigns [20]. As a result, the annual number of suspected and confirmed rabies cases in dogs has decreased due to an enhanced awareness about the disease. This in turn has led to veterinary laboratories and veterinarians identifying unusual cases of animals displaying rabies symptoms. More cases (and isolations) of rabies-related lyssaviruses should occur in future – like the cases described here. Identification of infections caused by rabies-related viruses is important and any additional information may improve our understanding of the epidemiology of these viruses. However, the recommended diagnostic technique for rabies, the FAT, cannot distinguish between the lyssavirus species. A broad-spectrum polyclonal fluorescein isothiocyanate conjugated anti-lyssavirus globulin, capable of detecting all lyssavirus species, is used in countries with laboratory diagnostics. In some MOKV cases atypical staining, i.e. the inclusions stain less intensely than is usually seen with rabies virus, has been observed [21]; as described for sample 14/024. The identification of a MOKV infection prompted a small retrospective study to determine if other MOKV cases, which did not produce atypical staining with the FAT, were overlooked. Subsequently, 35 samples collected over a 7 year period were tested and two additional MOKV cases from 2012 were identified. The remaining 33 samples were shown to belong to the canid variant of RABV.

In southern Africa, two variants of RABV circulate i.e. the canid variant infecting members of the *Canidae* family and the mongoose variant infecting members of the *Herpestidae* [22]. The conserved N-gene used in the Bayesian analysis is not ideally suited to distinguish between closely related viral variants. Nevertheless, the RABV sequences determined in this study all grouped with other canid variant RABV sequences from the same time period which is indicative of the continued rabies epidemical cycle in domestic dogs that was established in 1976 [23].

Bayesian analysis including all known MOKV isolates demonstrated a pattern of grouping of viruses according to geographical origin, irrespective of the gene(s) used; in agreement with previous reports [9]. The South African isolates grouped according to province – KZN and EC cluster. All previous isolates from KZN collected over a 28 year period (1970–1998) displayed a variation of 2.3% and 0.7% at the nucleotide and amino acid levels of the N-gene respectively. When comparing the new isolates from 2012–2014 (14 years after the last isolation from KZN) to the previous isolates from KZN, variation of 3% and 1.1% is observed at the nucleotide and amino acid levels of the N-gene respectively. Although the monophyletic grouping of South African MOKV isolates is indicative of the continual presence and stability of MOKV [9], the new isolates form a unique and well-supported clade (posterior probability = 1) within the KZN cluster. These new cases provide yet further confirmation that MOKV cycles are well established and that cases are underreported given the lack of capability/capacity to routinely characterize rabies positive samples and the lack of rabies surveillance in most developing countries. While the majority of MOKV cases are reported in cats, the identity of the reservoir species is unknown. It has been suggested that the reservoir might be a species that interacts with cats [9, 21], but there is little evidence to support this notion. MOKV has been isolated from shrews on three occasions and once from a rodent (*Lophuromys sikapusi*) and in conjunction with limited pathogenicity studies demonstrating significant amounts of virus in the saliva of shrews and rodents sufficient for transmission, invite speculation that these animals could be possible reservoir hosts [12, 24]. The majority of the lyssaviruses have a strong association with bats, with the exceptions of MOKV and Ikoma lyssavirus [25]. Given the diversity observed for bat lyssaviruses, it has been suggested that bats constitute the main evolutionary hosts [26]. Although MOKV has never been isolated from bats, the possibility of an African bat(s) reservoir cannot be excluded. Nevertheless, the identity of the reservoir host(s) for MOKV remains speculative. The close association of humans with domestic cats, an unknown reservoir and the lack of MOKV-protective vaccines [9, 11, 12] collectively support the argument that more research into the epidemiological aspects of MOKV is warranted.

## Conclusion

Considering the close contact of domestic cats with humans and the lack of protection from RABV based commercial vaccines, the risk to public and veterinary health is highlighted. These cases emphasize the lack of surveillance for rabies-related lyssaviruses and as such, the true incidence may be underestimated and is a major contributor to our incomplete understanding of the epidemiology and ecology of MOKV.

## Additional files

Additional file 1: Table S1. Primers and PCR conditions for amplification of the Nucleoprotein-, Phosphoprotein-, Matrix protein- and Glycoprotein genes of Mokola virus isolates. (XLSX 12 kb)
Additional file 2: Table S2. Details of sequences used for the Bayesian analysis of the rabies virus positive samples. (XLSX 11 kb)
Additional file 3: Table S3. Details of sequences used for the Bayesian analysis of the new Mokola virus isolates. (XLSX 11 kb)
Additional file 4: Figure S1. Bayesian analysis of the coding region of the Nucleoprotein gene (1353 bp) of all Mokola virus isolates (Additional file 3: Table S3) applying the general time reversible substitution model with invariable sites. Laboratory reference numbers are shown for all sequences, followed by the host species, country of origin (KZN SA: KwaZulu-Natal province, South Africa; EC SA: Eastern Cape province South Africa; ZIM: Zimbabwe; CAR: Central African Republic; NIG: Nigeria) and year of isolation. (TIFF 248 kb)
Additional file 5: Figure S2. Bayesian analysis of the coding region of the Phosphoprotein gene (913 bp) applying the general time reversible substitution model with gamma distribution. Laboratory reference numbers are shown for all sequences, followed by the host species, country of origin (KZN SA: KwaZulu-Natal province, South Africa; EC SA: Eastern Cape province South Africa; ZIM: Zimbabwe; CAR: Central African Republic; NIG: Nigeria) and year of isolation. (TIFF 222 kb)
Additional file 6: Figure S3. Bayesian analysis of the coding region of the Matrix protein gene (609 bp) applying the general time reversible substitution model with gamma distribution. Laboratory reference numbers are shown for all sequences, followed by the host species, country of origin (KZN SA: KwaZulu-Natal province, South Africa; EC SA: Eastern Cape province South Africa; ZIM: Zimbabwe; CAR: Central African Republic; NIG: Nigeria) and year of isolation. (TIFF 140 kb)
Additional file 7: Figure S4. Bayesian analysis of the coding region of the Glycoprotein gene (1569 bp) applying the general time reversible substitution model with gamma distribution and invariable sites. Laboratory reference numbers are shown for all sequences, followed by the host species, country of origin (KZN SA: KwaZulu-Natal province, South Africa; EC SA: Eastern Cape province South Africa; ZIM: Zimbabwe; CAR: Central African Republic; NIG: Nigeria) and year of isolation. (TIFF 218 kb)
Additional file 8: Table S4. Nucleotide identity of the Nucleoprotein gene of all Mokola virus isolates. (XLSX 10 kb)
Additional file 9: Table S5. Nucleotide identity of the Phosphoprotein gene of all Mokola virus isolates. (XLSX 10 kb)
Additional file 10: Table S6. Nucleotide identity of the Matrix protein gene of all Mokola virus isolates. (XLSX 10 kb)
Additional file 11: Table S7. Nucleotide identity of the Glycoprotein gene of all Mokola virus isolates. (XLSX 10 kb)

## Abbreviations

ARC-OVI: Agricultural research council-onderstepoort veterinary institute
EC: Eastern Cape province
FAT: Fluorescent antibody test
G-gene: Glycoprotein gene
KZN: KwaZulu-Natal province
MCMC: Markov Chain Monte Carlo
M-gene: Matrix protein gene
MOKV: Mokola virus
N-gene: Nucleoprotein gene
P-gene: Phosphoprotein gene
RABV: Rabies virus
VNAs: Virus neutralizing antibodies

## References

[1] Afonso CL, Amarasinghe GK, Bányai K, Bào Y, Basler CF, Bavari S, et al. Taxonomy of the order *Mononegavirales*: update 2016. Arch Virol. In Press. 10.1007/s00705-016-2880-1 PMC4947412 27216929

[2] Shope RE, Murphy FA, Harrison AK, Causey OR, Kemp GE, Simpson DIH, Moore DL (1970). Two African viruses serologically and morphologically related to rabies virus. J Virol.

[3] Kemp GE, Causey OR, Moore DL, Odelola A, Fabiyi A (1972). Mokola virus: Further studies on IbAn 27377, a new rabies-related etiological agent of zoonosis in Nigeria. Am J Trop Hyg.

[4] Familusi JB, Moore DL (1972). Isolation of a rabies-related virus from the cerebrospinal fluid of a child with aseptic meningitis. Afr J Med Sci.

[5] Familusi JB, Osunkoya BO, Moore DL, Kemp GE, Fabiyi A (1972). A fatal human infection with Mokola virus. Am J Trop Med Hyg.

[6] Jackson AC (2013). Current and future approaches to the therapy of human rabies. Antiviral Res.

[7] Weyer J, Msimang-Dermaux V, Paweska JT, le Roux K, Govender P, Coertse J (2016). A case of human survival of rabies, South Africa. S Afr J Infect Dis.

[8] Johnson N, Cunningham AF, Fooks AR (2010). The immune response to rabies virus infection and vaccination. Vaccine.

[9] Kgaladi J, Wright N, Coertse J, Markotter W, Marston D, Fooks AR (2013). Diversity and epidemiology of Mokola virus. PLoS Negl Trop Dis.

[10] Meredith CD, Nel LH, Von Teichman BF (1996). Further isolation of Mokola virus in South Africa. Vet Record.

[11] Foggin CM (1982). Atypical rabies virus in cats and a dog in Zimbabwe. Vet Record.

[12] Foggin CM (1988). Rabies and rabies-related viruses in Zimbabwe: Historical, virological and ecological aspects.

[13] Dean DJ, Abelseth MK, Atansiu P, Meslin FC, Kaplan MM, Koprowski H (1996). The fluorescent antibody test. Laboratory techniques in rabies.

[14] Coertse J, Weyer J, Nel LH, Markotter W (2010). Improved PCR methods for detection of African rabies and rabies-related lyssaviruses. J Clin Microbiol.

[15] Hall TA (1999). BioEdit: a user-friendly biological sequence alignment editor and analysis program for Windows 95/98/NT. Nucleic Acids Sym Ser.

[16] Darriba D, Taboada GL, Doallo R, Posada D (2012). jModelTest2: more models, new heuristics and high-performance computing. Nat Methods.

[17] Drummond AJ, Suchard MA, Xie D, Rambaut A (2012). Bayesian phylogenetics with BEUTi and the BEAST 1.7. Mol Biol Evol.

[18] Webster WA, Casey GA, Meslin FC, Kaplan MM, Koprowski H (1996). Virus isolation in neuroblastoma cell culture. Laboratory techniques in rabies.

[19] Markotter W, Kuzmin I, Rupprecht CE, Randles J, Sabeta CT, Wandeler AI (2006). Isolation of Lagos bat virus from water mongoose. Emerg Infect Dis.

[20] Nel LH (2013). Factors impacting the control of rabies. Microbiol Spec.

[21] Von Teichman BF, de Koker WC, Bosch SJE, Bishop GC, Meredith CD, Bingham J (1998). Mokola virus infection: description of recent South African cases and a review of the virus epidemiology. S Afr Vet Assoc.

[22] Von Teichman BF, Thomson GR, Meredith CD, Nel LH (1995). Molecular epidemiology of rabies virus in South Africa: evidence for two distinct virus groups. J Gen Virol.

[23] Coetzee P, Nel LH (2007). Emerging epidemic dog rabies in coastal South Africa: A molecular epidemiological analysis. Virus Res.

[24] Kemp GE, Moore DL, Isoun TT, Fabiyi A (1973). Mokola virus: Experimental infection and transmission studies with the shrew, a natural host. Arch Gesamte Virusforsch.

[25] Banyard AC, Evans JS, Luo TR, Fooks AR (2014). Lyssaviruses and bats: Emergence and zoonotic threat. Viruses.

[26] Rupprecht CE, Turmelle A, Kuzmin IV (2011). A perspective on lyssavirus emergence and perpetuation. Curr Opin Virol.

