# Supplementary figures and images for: New isolations of the rabies-related Mokola virus from South Africa
Source: BMC Vet Res. 2017 Jan 31;13:37. doi: 10.1186/s12917-017-0948-0 (PMC5282659; doi:10.1186/s12917-017-0948-0)

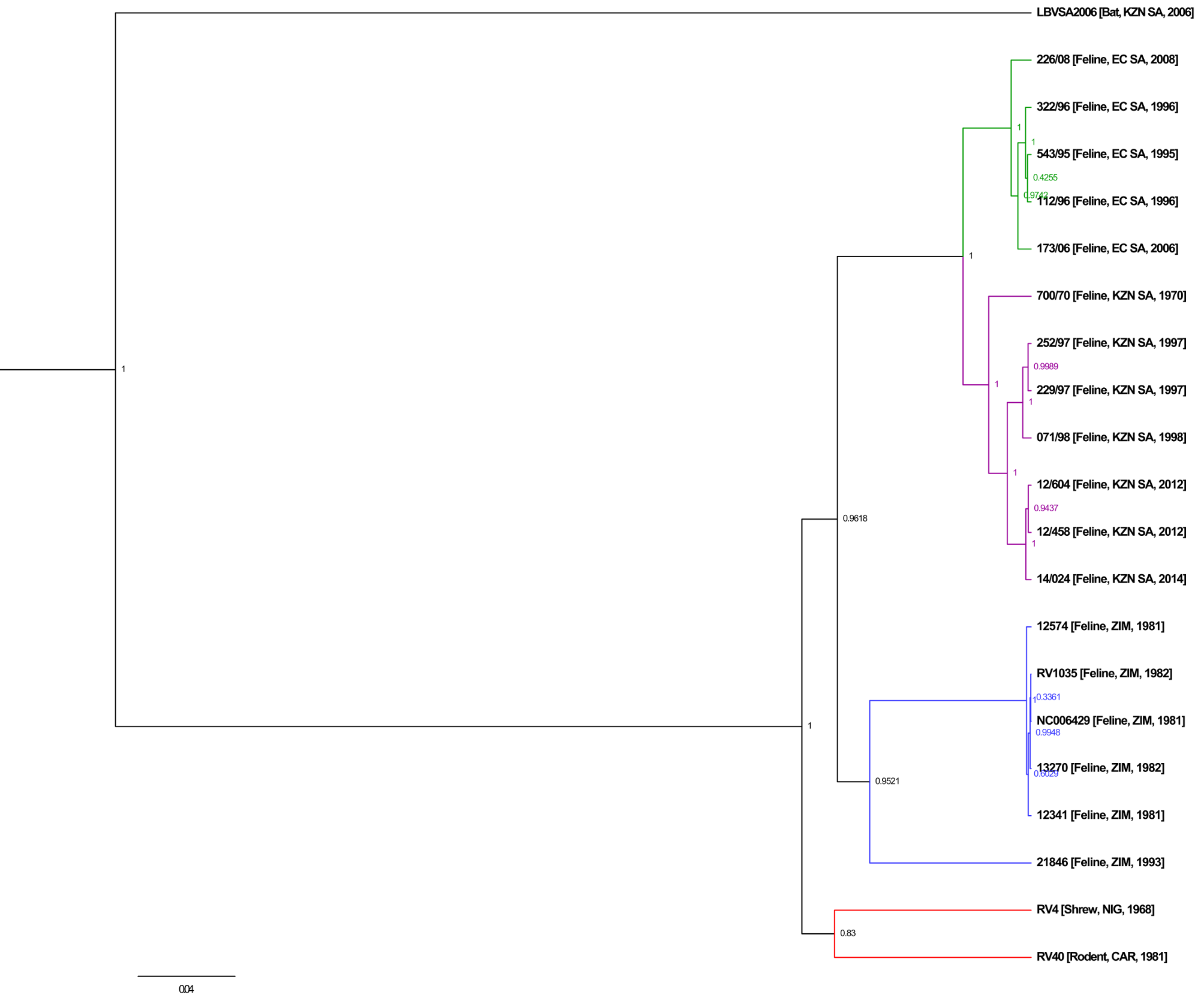

Supplement: Additional file 4: Figure S1. — Bayesian analysis of the coding region of the Nucleoprotein gene (1353 bp) of all Mokola virus isolates (Additional file 3: Table S3) applying the general time reversible substitution model with invariable sites. Laboratory reference numbers are shown for all sequences, followed by the host species, country of origin (KZN SA: KwaZulu-Natal province, South Africa; EC SA: Eastern Cape province South Africa; ZIM: Zimbabwe; CAR: Central African Republic; NIG: Nigeria) and year of isolation. (TIFF 248 kb) [file 12917_2017_948_MOESM4_ESM.tiff]

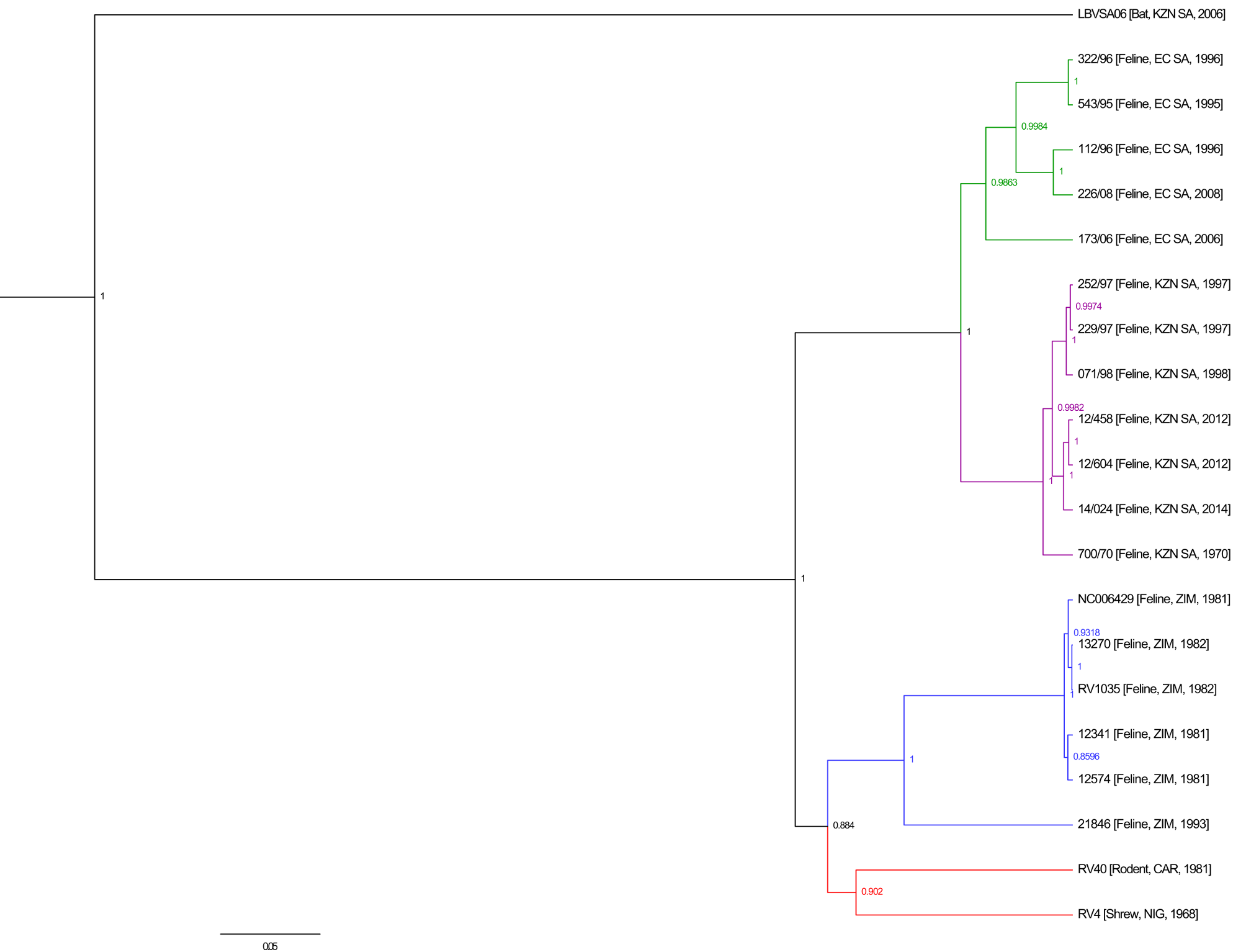

Supplement: Additional file 5: Figure S2. — Bayesian analysis of the coding region of the Phosphoprotein gene (913 bp) applying the general time reversible substitution model with gamma distribution. Laboratory reference numbers are shown for all sequences, followed by the host species, country of origin (KZN SA: KwaZulu-Natal province, South Africa; EC SA: Eastern Cape province South Africa; ZIM: Zimbabwe; CAR: Central African Republic; NIG: Nigeria) and year of isolation. (TIFF 222 kb) [file 12917_2017_948_MOESM5_ESM.tiff]

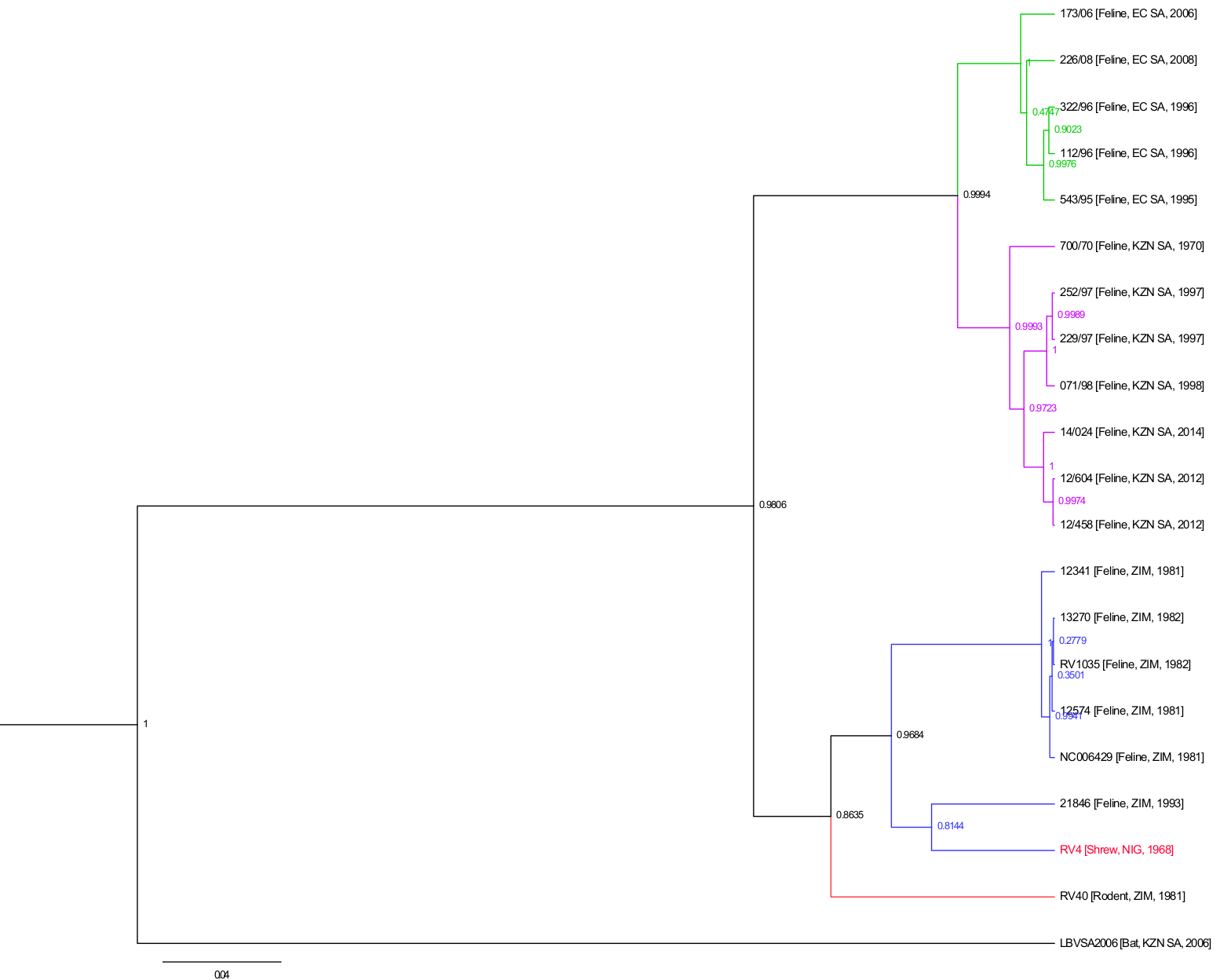

Supplement: Additional file 6: Figure S3. — Bayesian analysis of the coding region of the Matrix protein gene (609 bp) applying the general time reversible substitution model with gamma distribution. Laboratory reference numbers are shown for all sequences, followed by the host species, country of origin (KZN SA: KwaZulu-Natal province, South Africa; EC SA: Eastern Cape province South Africa; ZIM: Zimbabwe; CAR: Central African Republic; NIG: Nigeria) and year of isolation. (TIFF 140 kb) [file 12917_2017_948_MOESM6_ESM.tiff]

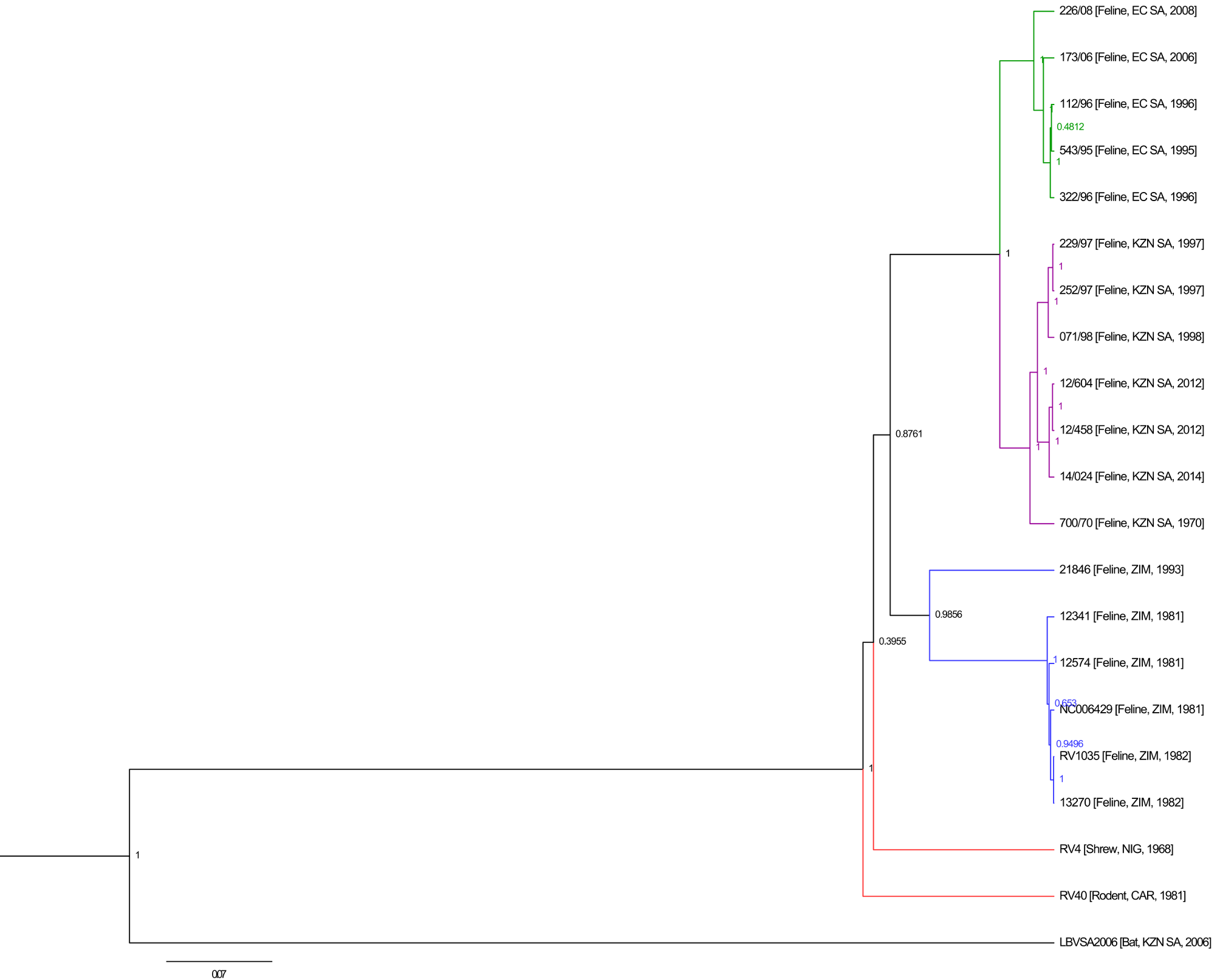

Supplement: Additional file 7: Figure S4. — Bayesian analysis of the coding region of the Glycoprotein gene (1569 bp) applying the general time reversible substitution model with gamma distribution and invariable sites. Laboratory reference numbers are shown for all sequences, followed by the host species, country of origin (KZN SA: KwaZulu-Natal province, South Africa; EC SA: Eastern Cape province South Africa; ZIM: Zimbabwe; CAR: Central African Republic; NIG: Nigeria) and year of isolation. (TIFF 218 kb) [file 12917_2017_948_MOESM7_ESM.tiff]
